# Intelligent GelMA microspheres co-loaded with BMSCs and Kartogenin for spatiotemporally controlled release and immunomodulatory regeneration of temporomandibular joint osteoarthritis

**DOI:** 10.3389/fimmu.2026.1921455

**Published:** 2026-09-16

**Authors:** Feng Feng, Bin Xing, Wen Liu

**Affiliations:** 1 Department of Stomatology, Qingdao Hospital, University of Health and Rehabilitation Sciences(Qingdao Municipal Hospital), Qingdao, Shandong, China; 2 Department of Stomatology, Qingdao Women and Children’s Hospital, Qingdao University, Qingdao, Shandong, China; 3 Department of Stomatology, Qilu Hospital (Qingdao), Cheeloo College of Medicine, Shandong University, Qingdao, Shandong, China

**Keywords:** BMSCs, cartilage regeneration, immunomodulatory microsphere, spatiotemporally controlled, temporomandibular joint osteoarthritis

## Abstract

**Objective:**

This study aims to overcome the limitations of current treatments for temporomandibular joint osteoarthritis (TMJOA), which often fail to promote cartilage regeneration and necessitate repeated administrations. To this end, an injectable intelligent GelMA microsphere platform co-loaded with bone marrow mesenchymal stem cells (BMSCs) and the small molecule Kartogenin (KGN) was developed. This system facilitates spatiotemporally controlled release and immunomodulatory regeneration within the joint cavity. The therapeutic efficacy and underlying mechanisms of this approach for TMJOA were systematically evaluated.

**Methods:**

GelMA porous microspheres loaded with BMSCs and KGN were fabricated utilizing microfluidic technology. The physicochemical characteristics of the microspheres and the *in vitro* release profile of KGN were assessed through scanning electron microscopy, swelling assays, degradation studies, and high-performance liquid chromatography. *In vitro* biocompatibility was evaluated by co-culturing the microspheres with rabbit chondrocytes, followed by analysis of the expression of genes and proteins associated with chondrogenic differentiation. The anti-inflammatory effects were investigated via macrophage polarization assays. *In vivo*, a rabbit TMJOA model was established using sodium iodoacetate and subsequently divided into control, OA, GelMA@KGN microsphere, and GelMA@KGN-BMSC microsphere groups. Histological staining, immunohistochemistry, immunofluorescence, and qRT-PCR analyses of the condylar tissue were conducted at 4 and 8 weeks post-treatment.

**Results:**

The prepared GelMA@KGN-BMSC microspheres demonstrated uniform particle size, interconnected porosity, favorable swelling behavior, and characteristic degradation profiles, with KGN exhibiting a sustained release pattern. *In vitro* studies revealed a high viability of chondrocytes, with cells exhibiting extensive spreading on the microsphere surfaces. In the GelMA@KGN-BMSC group, chondrocytes showed significantly elevated expression levels of chondrogenic-related genes and proteins, alongside a marked reduction in the expression of matrix-degrading enzymes. Macrophage polarization assays indicated that these microspheres substantially promoted M2 polarization while inhibiting M1 polarization. *In vivo* assessments revealed that treatment with the GelMA@KGN-BMSC microspheres preserved cartilage structure integrity, significantly enhanced cartilage matrix synthesis and reduced inflammatory factor expression. These therapeutic effects were notably superior to those observed in the GelMA@KGN microsphere group.

**Conclusion:**

The GelMA@KGN-BMSC intelligent microsphere system developed in this study enables localized delivery of BMSCs and sustained release of KGN through a single intra-articular injection. These GelMA@KGN-BMSC microspheres significantly enhance cartilage regeneration via a synergistic mechanism that integrates induced differentiation and immunomodulation, offering a safe and effective minimally invasive therapeutic approach for TMJOA.

## 1 Introduction

Temporomandibular joint osteoarthritis (TMJOA) is a degenerative joint disorder predominantly manifested by mandibular movement impairments, joint noises, and escalating pain (1–3). The pathogenesis of TMJOA is multifactorial, with synovial inflammation and chondrocyte dysfunction identified as the principal factors aggravating disease progression (4–7). Within the synovial tissue of the affected joint, macrophages and fibroblast-like synoviocytes exhibit abnormal activation, leading to the secretion of elevated levels of pro-inflammatory cytokines, including tumor necrosis factor-alpha (TNF-α), interleukin-1 beta (IL-1β) and interleukin-6 (IL-6) (8–11). These inflammatory mediators directly stimulate chondrocytes to produce matrix metalloproteinases, such as MMP-13, thereby accelerating the degradation of type II collagen (COL-2) and aggrecan (ACAN) (12, 13). The regulation of chondrocyte fate in TMJOA involves the aberrant activation of multiple forms of programmed cell death, including chondrocyte apoptosis, ferroptosis and dysregulation of autophagy (14–17). These processes collectively result in a pronounced imbalance between cartilage matrix synthesis and degradation. Furthermore, the avascular and non-neural nature of articular cartilage severely limits its intrinsic capacity for self-repair (18). Consequently, degenerated cartilage is notably difficult to regenerate, rendering the clinical management of TMJOA a particularly challenging issue.

Current clinical treatment strategies for TMJOA can be categorized into three main approaches, including non-invasive treatments, minimally invasive treatments and surgical interventions. Non-invasive approaches encompass the use of non-steroidal anti-inflammatory drugs, physical therapy and occlusal splint therapy (7, 19–21). While these methods provide temporary relief from pain and inflammation, they do not halt or reverse cartilage degeneration. Minimally invasive treatments include joint lavage and intra-articular injections of biological agents such as hyaluronic acid or platelet-rich plasma (22–24). These interventions are limited by factors such as short drug half-life, the necessity for multiple repeated injections and uncertain long-term efficacy. Surgical options including joint replacement and condylectomy are highly invasive, costly and generally reserved for patients with end-stage TMJOA (25). Consequently, there is a critical need to develop novel therapeutic strategies that can concurrently inhibit inflammation, promote cartilage regeneration and ensure sustained intra-articular retention, thereby addressing the limitations of current treatments.

In recent years, stem cell-based regenerative medicine has emerged as a promising approach for the treatment of TMJOA (26–28). Among the various cell types, bone marrow-derived mesenchymal stem cells (BMSCs) have gained prominence due to their robust chondrogenic differentiation capacity, immunomodulatory properties and relative ease of isolation (29). BMSCs are capable of differentiating into chondrocytes, thereby contributing to the repair of damaged cartilage matrix. Additionally, BMSCs secrete anti-inflammatory cytokines and growth factors via paracrine mechanisms, which help modulate the inflammatory microenvironment within the joint cavity (30, 31). However, the direct intra-articular injection of free BMSCs presents significant challenges, such as low cell survival rates, limited residence time within the joint and uncontrollable differentiation pathways (32, 33). These limitations substantially hinder the clinical effectiveness of this approach. To address these issues, recent research has focused on the combined application of small molecule compounds to precisely direct the differentiation fate of BMSCs. Among these compounds, Kartogenin (KGN), a small heterocyclic molecule exhibits distinct advantages that has garnered significant attention in recent years. KGN performs dual functions by promoting chondrogenic differentiation and protecting cartilage tissue (34–36). In recent studies, KGN facilitates the directed differentiation of mesenchymal stem cells into chondrocytes through the activation of the CBFβ-RUNX1 signaling pathway (37). Concurrently, KGN activates the NRF2 antioxidant pathway by suppressing the expression of miR-146a, which leads to the downregulation of matrix-degrading enzymes such as matrix metalloproteinase 13 (MMP13) and ADAMTS5 (38). This reduction in enzyme expression mitigates cartilage matrix degradation, thereby conferring a pronounced cartilage-protective effect. Therefore, the combined application of KGN and BMSCs holds promise for overcoming the challenge of uncontrollable differentiation direction in cell therapy. However, direct intra-articular injection of either free BMSCs or free KGN is limited by rapid clearance from the joint space, which hinders prolonged retention at the lesion site and sustained therapeutic effects. To address this issue, we propose co-encapsulating BMSCs and KGN within an appropriate delivery vehicle to achieve localized cell retention and controlled, sustained drug release following a single intra-articular injection. This strategy aims to simultaneously overcome three critical challenges, low cell survival rate, uncontrollable differentiation and rapid drug clearance. According to this, they will maximize the synergistic therapeutic potential of combining pharmacological agents with tissue engineering approaches.

Therefore, the development of appropriate drug delivery carriers to enable the sustained release of KGN is essential for maximizing its therapeutic potential. Gelatin methacryloyl (GelMA) is a photo-crosslinkable hydrogel characterized by excellent biocompatibility, adjustable mechanical properties and degradation rates, making it widely utilized in cartilage tissue engineering. Notably, GelMA porous microspheres fabricated via microfluidic technology offer advantages including uniform size distribution, enhanced injectability and porous architectures that promote cell adhesion and nutrient exchange. Consequently, GelMA microspheres represent an ideal three-dimensional scaffold for BMSCs, capable of effectively loading KGN and facilitating its sustained release.

Building upon the aforementioned background, this study proposes a tissue engineering treatment strategy focused on BMSCs. We employed microfluidic technology to fabricate porous GelMA microspheres, which were concurrently loaded with rabbit BMSCs and KGN (GelMA@KGN-BMSC) or loaded with KGN alone (GelMA@KGN). These formulations were administered via a single intra-articular injection to treat TMJOA. This study addresses the following scientific questions. Firstly, it is necessary to determine whether GelMA microspheres offer an appropriate three-dimensional microenvironment for BMSCs, thereby enhancing cell viability. Secondly, the potential of sustained release of KGN from the microspheres to specifically induce the differentiation of BMSCs into chondrocytes, as well as to exert anti-inflammatory and cartilage-protective effects should be evaluated. Finally, it is important to assess whether BMSCs can synergize with KGN to promote cartilage regeneration, inhibit extracellular matrix degradation and modulate the inflammatory microenvironment, ultimately contributing to the effective reversal of TMJOA progression.

To address the issues outlined above, this study utilized a sodium iodoacetate-induced rabbit model of TMJOA, establishing four experimental groups, including a control group, an osteoarthritis (OA) group, a GelMA@KGN group, and a GelMA@KGN-BMSC group. Through comprehensive and systematic *in vitro* cellular assays and *in vivo* animal experiments, the biocompatibility, chondrogenic induction capacity and therapeutic efficacy of these microspheres in mitigating cartilage degeneration were evaluated. The GelMA@KGN group was included to elucidate the specific contribution of BMSCs in the treatment process. By comparing therapeutic outcomes between the GelMA@KGN and GelMA@KGN-BMSC groups, the study demonstrated the advantages of a synergistic approach combining pharmacological agents with tissue engineering techniques over drug treatment alone. This research aims to develop a safe, effective and minimally invasive tissue engineering strategy capable of being administered as a single treatment for the clinical management of TMJOA.

## 2 Materials and methods

### 2.1 Preparation of GelMA@KGN-BMSC

#### 2.1.1 Preparation of KGN/β-CD inclusion complex

The appropriate amount of KGN was weighed and dissolved in anhydrous ethanol, while β-cyclodextrin (β-CD) was dissolved in deionized water. Under magnetic stirring, the KGN solution was gradually added dropwise to the β-CD solution and stirred at room temperature in the dark for 24 hours. Subsequently, the ethanol was removed by rotary evaporation, and the resulting mixture was freeze-dried to yield a white powdery KGN/β-CD inclusion complex.

#### 2.1.2 Extraction and culture of rabbit BMSCs

Four week-old health New Zealand white rabbits were anesthetized via injection of pentobarbital sodium into the marginal ear vein. Using the posterior superior iliac spine as the puncture site, a needle was inserted perpendicularly through the bone cortex until a distinct breakthrough sensation was perceived. The needle core was then withdrawn and connected to a sterile syringe containing 100 U/mL heparin sodium to aspirate 5 mL of bone marrow. Subsequently, the aspirated bone marrow was promptly transferred into a centrifuge tube containing low-glucose Dulbecco’s Modified Eagle Medium (DMEM) supplemented with 10% fetal bovine serum (FBS) and 1% double antibiotics for further use.

Carefully overlay the collected bone marrow fluid with an equal volume of complete culture medium, then centrifuge at 1800 rpm for 5 minutes. Discard the supernatant and repeat this procedure three times. Subsequently, resuspend the cell pellet in complete culture medium, adjust the cell density and seed the cells into 10 cm culture dishes. Incubate the cultures at 37 °C in a 5% CO_2_ atmosphere. After three days, wash the cultures with culture medium to remove non-adherent cells and discard these cells. Replace with fresh complete culture medium. When the cells reach approximately 80% confluence, digest them using 0.25% trypsin-EDTA and passage at a ratio of 1:3. Cells at the third passage are utilized for subsequent experiments.

#### 2.1.3 Preparation of microspheres

The GelMA utilized in this study is a commercially available product obtained from Huaxia Siyi (Shanghai) Biotechnology Co., Ltd., and the product number R201B2. To fabricate GelMA@KGN-BMSC microspheres, a 10% (w/v) GelMA solution containing 0.25% (w/v) lithium phenyl-2, 4, 6-trimethylbenzoylphosphinate (LAP) photoinitiator was initially prepared and sterilized by filtration through a 0.22 μm membrane filter. Subsequently, the KGN/β-cyclodextrin inclusion complex was incorporated into this solution to achieve a final concentration of 5 μM and mixed thoroughly. BMSCs were harvested, washed with sterile phosphate-buffered saline (PBS), and centrifuged at 1800 rpm for 5 minutes. The supernatant was then discarded. The resulting cell pellet was resuspended in the prepared GelMA solution at a density of 5 × 10^7^ cells/mL and gently pipetted to ensure homogeneous mixing while minimizing bubble formation.

Microspheres were fabricated utilizing a flow-focusing microfluidic chip composed of polydimethylsiloxane, featuring channel dimensions of 150 μm in both width and height. Initially, the oil phase injection pump was activated, maintaining a flow rate of 20 μL/min. Once a stable and continuous flow of the oil phase was established within the microchannel, the water phase injection pump, containing a mixture of BMSCs, KGN, and GelMA, was initiated with the flow rate adjusted to 5 μL/min. The flow rate ratio of the water phase to the oil phase is 0.25, with a combined total flow rate of 25 μL/min. The shear forces at the interface between the water and oil phases facilitated the stable generation of monodisperse water phase droplets at the chip’s collection outlet, each uniformly encapsulating cells. These droplets were collected in a tube and immediately exposed to 405 nm ultraviolet light for one minute to trigger a LAP-mediated photoinitiated free radical polymerization, resulting in the crosslinking of GelMA and the formation of structurally stable GelMA@KGN-BMSC microspheres.

Subsequently, a demulsifier was added to the collection tube at a volume ratio of 1:1, followed by gentle inversion to ensure thorough mixing. The mixture was then allowed to stand for 5 minutes to facilitate the complete release of microspheres from the oil phase into the lower aqueous phase. The upper oil phase and demulsifier mixture were carefully aspirated, and the lower aqueous solution containing the microspheres was collected. An appropriate volume of physiological saline was added to the collected solution, which was gently resuspended and then subjected to low-speed shaking for 1 minute. The solution was subsequently centrifuged at 800 rpm for 3 minutes, and the supernatant was discarded. These washing steps were repeated three times to effectively remove residual oil phase, demulsifier, and un-crosslinked GelMA monomers. Ultimately, GelMA@KGN-BMSC microspheres with a cell encapsulation density of no less than 5 × 10^7^ cells/mL were obtained.

Pure GelMA microspheres were prepared using only an aqueous phase comprising GelMA solution delivered through an injection pump. In contrast, GelMA@KGN microspheres were fabricated using a mixture containing both KGN and GelMA. All other preparation procedures were conducted in accordance with the previously described method.

### 2.2 Material characterization of GelMA@KGN-BMSC

#### 2.2.1 Morphology and particle size

Freshly prepared microspheres were collected, washed with PBS and subsequently freeze-dried. Following gold sputtering, the surface morphology of the microspheres was observed using a scanning electron microscope (SEM, ZEISS Sigma 360, Germany) operated at an accelerating voltage of 3 kV. Concurrently, a minimum of 50 pores were randomly selected from each group of microspheres, and their diameters were measured using ImageJ software. Subsequently, statistical analyses were performed to determine the range and mean of the pore size distribution.

Additionally, fresh wet microspheres were placed in a culture dish, and a minimum of 100 microspheres were randomly imaged using an optical microscope. The diameters of the microspheres were measured with ImageJ software to determine the average particle size and particle size distribution.

#### 2.2.2 Swelling performance

Weigh Equal masses of freeze-dried GelMA microspheres, GelMA@KGN, and GelMA@KGN-BMSC designated respectively as W_d1, W_d2, and W_d3. These microspheres were then immersed in PBS at pH 7.4 and 37 °C, with samples removed at 1, 2, 4, 6, 8, 10, 12 hours. After gently blotting surface moisture using filter paper, the samples were weighed again, recorded as W_w1, W_w2, and W_w3. Once the weights stabilized, the final measurements were used to calculate the swelling ratio. Each group included three parallel samples.


Swelling Ratio (%)=(W_w−W_d)W_d×100%

#### 2.2.3 Degradation evaluation *in vitro*

Equal masses of freeze-dried GelMA microspheres, GelMA@KGN, and GelMA@KGN-BMSC were weighed and recorded as W_0. Each type of microsphere was separately placed into PBS containing 1 U/mL collagenase type I and incubated at 37 °C on a shaking incubator set to 100 rpm. At predetermined time points (1, 3, 7, 14, 21, and 28 days), the microspheres were retrieved. Following freeze-drying, the samples from the three groups were weighed and recorded respectively as W_t1, W_t2, and W_t3. The degradation rates of the three groups of microspheres were then calculated based on the initial mass W_0.


Degradation Rate (%)=W_tW_0×100%

#### 2.2.4 Mechanical properties

Dynamic oscillation tests were performed using a rheometer (TA Instruments, DHR-2). Three groups of microspheres were uniformly distributed on parallel plates with a diameter of 20 mm, maintaining a gap of 1 mm. Frequency scans were conducted at 25 °C with a strain of 1% over a frequency range of 0.1 to 10 Hz, during which the storage modulus (G’) and loss modulus (G’’) were recorded. Three samples were tested for each group.

#### 2.2.5 Release rate testing of KGN *in vitro*

10mg of GelMA@KGN and GelMA@KGN-BMSC were placed into dialysis bags with a molecular weight cutoff of 14 kDa. Each bag was filled with 2 mL of PBS (pH 7.4) containing 0.1% Tween-80, serving as the release medium. After sealing, the dialysis bags were transferred into 15 mL centrifuge tubes and incubated in a constant temperature shaker at 37 °C with agitation at 100 rpm. At predetermined time points (days 1, 3, 5, 7, 10, 14, 21, and 28), the entire release medium was collected and replaced with an equal volume of fresh medium. The concentration of KGN in the collected release medium was quantified using HPLC, and a cumulative release profile was generated as a function of time.

#### 2.2.6 Proliferation assay of BMSCs within microspheres

The prepared GelMA@KGN-BMSC microspheres were gently washed twice with sterile PBS and subsequently resuspended in complete culture medium. Approximately 50 microspheres were seeded into each well of a 96-well plate. Subsequently, 100 μL of complete culture medium was added to each well, and the plate was incubated at 37 °C in an atmosphere containing 5% CO_2_. At 1, 3, and 7 days of culture, 10 μL of CCK-8 reagent was added to each well, followed by incubation at 37 °C in the dark for 2 hours. After incubation, the absorbance (optical density, OD) was measured at 450 nm using a microplate reader. Wells containing only culture medium and CCK-8 reagent, without microspheres, served as blank controls to account for background interference. Each experimental group included five replicates, and the entire experiment was independently repeated three times.

### 2.3 Biocompatibility evaluation of GelMA@KGN-BMSC *in vitro*

#### 2.3.1 Isolation and culture of rabbit chondrocytes

To more precisely elucidate the effects and functional impact of microspheres on target cells, chondrocytes were selected for subsequent biocompatibility assessment. Healthy 4-week-old New Zealand White rabbits were anesthetized by intravenous administration of pentobarbital sodium through the auricular vein. A longitudinal incision was made along the inner side of the rabbit ear, and the subcutaneous tissue was bluntly dissected to fully expose and peel off the perichondrium from the auricular cartilage. Subsequently, auricular cartilage slices were harvested. These cartilage slices were placed in PBS containing 1% penicillin-streptomycin and rinsed three times to remove residual blood and soft tissue. Using a sterile surgical blade, the cartilage was then sectioned into tissue blocks approximately 1 mm³ in size.

Transfer the minced cartilage tissue into a centrifuge tube containing 0.15% type II collagenase and incubate overnight at 37 °C with agitation at 100 rpm using a shaking incubator. Following digestion, filter the suspension through a 200-mesh cell strainer and collect the filtrate. Centrifuge the filtrate at 1600 rpm for 5 minutes, then discard the supernatant. Add high-glucose DMEM complete medium supplemented with 10% fetal bovine serum (FBS) and 1% double antibiotics, and seed the cells at a density of 1×10^7^ in a 10 cm culture dish. When the cells reach approximately 80% confluence, digest them with 0.25% trypsin-EDTA and passage at a ratio of 1:3. Utilize cells at the third passage (P3) for subsequent experiments.

#### 2.3.2 Cell viability assay

A Transwell non-contact co-culture system was employed to assess the impact of microspheres on chondrocyte proliferation. P3 chondrocytes were seeded at a density of 1 × 10^4^ cells per well in the lower chamber of a 24-well plate and allowed to adhere overnight. Subsequently, GelMA microspheres, GelMA@KGN microspheres, and GelMA@KGN-BMSC microspheres (approximately 50 microspheres per well) were introduced into the Transwell inserts, which were then placed into the corresponding wells to facilitate indirect co-culture between the microspheres and chondrocytes. A control group consisting of chondrocytes cultured without microspheres was included as a blank control. At day 7 of culture, the samples were washed with PBS. Subsequently, Live/Dead staining was conducted by applying a working solution containing 2 μM Calcein-AM and 4 μM propidium iodide, followed by incubation at 37 °C in the dark for 15 minutes. Observations and imaging were performed using a laser confocal microscope. Live cells exhibited green fluorescence, while dead cells displayed red fluorescence. The proportion of live cells was quantified using ImageJ software.

#### 2.3.3 Cell proliferation assay

The Transwell non-contact co-culture system was employed to assess the impact of microspheres on chondrocyte proliferation. The detailed methodology is provided in Section 2.3.2. On days 1, 4, and 7 of culture, the Transwell inserts were removed, and 10 μL of CCK-8 reagent was added to each well in the lower chamber. Following incubation at 37 °C for 2 hours, absorbance at 450 nm OD was measured using a microplate reader. The OD value of the blank control group was designated as 100%, and the relative proliferation rate of cells in each experimental group was calculated accordingly.

#### 2.3.4 Cell adhesion and morphology

After co-culturing the aforementioned microsphere groups with P3 chondrocytes in 6-well plates for 24 hours, the cells were washed with PBS and fixed using 4% paraformaldehyde. Subsequently, the cells were permeabilized with 0.2% Triton X-100 and blocked with 1% bovine serum albumin (BSA). Fluorescein isothiocyanate (FITC)-labeled phalloidin and 4’, 6-diamidino-2-phenylindole (DAPI) staining were then performed to observe the cytoskeleton and nuclei under a confocal microscope. Concurrently, conduct Live/Dead staining following the protocol outlined in Section 2.3.2. Furthermore, cells and microspheres were co-cultured in suspension at equivalent densities within low-adhesion culture dishes for a duration of three days. The adhesion of cells to the microspheres was subsequently examined using both optical microscopy and confocal microscopy.

### 2.4 Functional evaluation of GelMA@KGN-BMSC

#### 2.4.1 Function of chondrogenic

To evaluate the role of microspheres in ECM secretion by chondrocytes, an inflammation-induced chondrocyte injury model was established to determine whether GelMA@KGN-BMSC can mitigate or reverse ECM degradation caused by inflammation. Following chondrocyte seeding, the culture medium was replaced the next day with complete medium supplemented with 10 ng/mL interleukin-1 beta (IL-1β) to induce inflammation. Concurrently, GelMA@KGN and GelMA@KGN-BMSC microspheres were co-cultured in each experimental group as previously described. Additionally, a group of normal chondrocytes cultured without induction was established as the blank control, while a group of chondrocytes subjected solely to induction served as the IL-1β control. Following co-culture of the aforementioned microsphere groups with P3 chondrocytes in Transwell chambers for a day and 3 days, total RNA was isolated from the chondrocytes utilizing an RNA extraction kit. The extracted RNA was then reverse transcribed into complementary DNA (cDNA) employing a reverse transcription kit. The primer sequences applied in this study are provided in **Table 1**. Quantitative real-time PCR (qRT-PCR) was conducted using the SYBR Green method to assess the expression levels of genes including SOX9, COL2A1, ACAN, MMP13, ADAMTS5, TNF-α, and iNOS (inducible nitric oxide synthase). Glyceraldehyde 3-phosphate dehydrogenase (GAPDH) served as the internal control, and relative gene expression was quantified using the 2-ΔΔCt method. All results were normalized with respect to the measurements obtained on the first day in Blank group.

**Table 1:** Primer sequences used for qRT‑PCR analysis.

Gene	Forward (5’→3’)	Reverse (5’→3’)
COL2A1	ATGGCGGCTTCCACTTCA	CTCAGTGGACAGCAGGCG
ACAN	TTACCACCTACCCTTCACCTG	TTCTTCTGTCCAAAGGTCCTG
SOX9	GGGAAGCTCTGGAGACTGCT	TGTAGTCCGGGTGGTCTTTC
MMP13	GCCATTCCTTAGGGCTTGA	GCCATTCCTTAGGGCTTGA
ADAMTS5	ACTACGATGCAGCTATCCTGT	ACTACGATGCAGCTATCCTGT
TNF-α	ACTACGATGCAGCTATCCTGT	CTACGTGGGCTAGAGGCTTG
iNOS	GTTCTCAGCCCAACAATACAAGA	GTTCTCAGCCCAACAATACAAGA
Arg-1	CTCCAAGCCAAAGTCCTTAGAG	CTCCAAGCCAAAGTCCTTAGAG
CD206	TGGCAAGTATCCACAGCA	GGTTCCATCACTCCACTCA
IL-10	GCCCTTTGCTATGGTGTCCT	GCCCTTTGCTATGGTGTCCT

RIPA lysis buffer supplemented with 1% protease inhibitor was added to the cell samples co-cultured for 3 days and lysed on ice for 30 minutes. The lysates were then collected and centrifuged at 12, 000g for 15 minutes at 4 °C, and the supernatants were retained. Total protein concentration was determined using a BCA protein assay kit. Equal amounts of protein were mixed with 5× loading buffer and boiled for 10 minutes to achieve denaturation. Protein samples were separated by SDS-PAGE and subsequently transferred onto PVDF membranes by wet transfer. Membranes were blocked at room temperature with 5% non-fat milk for one hour, followed by overnight at 4 °C with primary antibodies against COL2A1, ACAN, SOX9, MMP13, ADAMTS5, TNF-α, iNOS, and the internal control GAPDH. The following day, membranes were washed three times with TBST for 10 minutes each. HRP-conjugated goat anti-rabbit IgG secondary antibody was applied and incubated at room temperature for one hour. After washing with TBST, membranes were developed using an ECL chemiluminescence reagent, and images were captured using a gel imaging system. Grayscale analysis was performed using ImageJ software, and relative protein expression levels were quantified as the ratio of target protein grayscale intensity to that of GAPDH.

#### 2.4.2 Analysis of macrophage polarization and anti-inflammatory

The mouse mononuclear macrophage leukemia cell line RAW 264.7 were maintained in high-glucose DMEM medium containing 10% FBS and 1% penicillin-streptomycin in a humidified incubator at 37 °C with 5% CO_2_. Following passaging, polarization was induced according to experimental protocols. RAW 264.7 cells were seeded into 24-well plates containing coverslips for immunofluorescence staining and into 6-well plates for ELISA and RT-qPCR assay. Following overnight adhesion to the wall, the cells were divided into six groups and co-cultured with the following treatments: (1) control group (model group), (2) GelMA microspheres group, (3) free KGN group (5 μM, equivalent to the KGN concentration loaded in the microspheres), (4) BMSCs supernatant group (conditioned medium collected from BMSCs cultured for 48 h), (5) GelMA@KGN microspheres group, and (6) GelMA@KGN-BMSC microspheres group. Concurrently, polarization treatment was conducted separately on RAW 264.7 cells. Specifically, for M1 polarization, cells were cultured in complete medium supplemented with 100 ng/mL lipopolysaccharide (LPS) and 20 ng/mL recombinant mouse interferon-gamma (IFN-γ). For M2 polarization, cells were cultured in complete medium containing 20 ng/mL recombinant mouse interleukin-4 (IL-4) and 20 ng/mL recombinant mouse interleukin-13 (IL-13).

For fluorescence staining, following a 24-hour co-culture period, the culture medium was discarded, and the samples were washed three times with PBS. Subsequently, cells were fixed with 4% paraformaldehyde at room temperature for 15 minutes. After washing with PBS, permeabilization was performed using 0.2% Triton X-100 at room temperature for 10 minutes. Samples were then blocked with 5% BSA at room temperature for 1 hour. Primary antibodies targeting iNOS, TNF-α, IL-1β, Arg-1 (Arginase-1), CD206, and IL-10 were applied individually and incubated overnight at 4 °C. The following day, samples were washed with PBS and incubated with the appropriate fluorescent secondary antibodies at room temperature in the dark for 1 hour. Finally, the slides were mounted using an anti-fade mounting medium containing DAPI, and fluorescence images were captured using a laser confocal microscope.

To detect the secretion of inflammatory factors, collect the supernatant after 48 hours of co-culture. Following the manufacturer’s instructions for the ELISA kit, measure the concentrations of M1-associated inflammatory factors including iNOS, TNF-α, and IL-1β, as well as M2-associated anti-inflammatory factors such as Arg-1, CD206, and IL-10. Use a microplate reader to measure absorbance at 450 nm, and determine the concentration of each factor by referencing the standard curve.

To detect the expression of polarization marker genes, RAW 264.7 cells were collected from 6-well plates after 48 hours of co-culture. Total RNA was extracted using TRIzol reagent and subsequently reverse transcribed into cDNA. qRT-PCR was performed employing the SYBR Green method to assess the mRNA expression levels of M1 and M2 markers, with GAPDH serving as the internal reference gene. Relative gene expression levels were calculated using the 2^-ΔΔCt^ method. All results were normalized with respect to the measurements obtained on Control group.

To elucidate the molecular mechanisms underlying macrophage polarization, this study employed Western blot analysis to assess the activation of the NF-κB signaling pathway. Cells were harvested, and the levels of phosphorylated p65 (p-p65) protein were quantified following established protocols. Grayscale value analysis was performed using ImageJ software, and the expression levels of p-p65 were normalized to GAPDH.

### 2.5 Modeling and treatment of TMJOA in rabbits

#### 2.5.1 Experimental animal grouping

This study utilized 24 healthy adult male New Zealand White rabbits, each weighing between 2.5 and 3.0 kg. All experimental procedures involving animals were approved by the Qilu Hospital (Qingdao) Experimental Animal Ethics Committee. Following one week acclimation period, the animals were randomly assigned to four groups, with six rabbits per group. The control group received standard feeding without intervention. The OA group underwent disease modeling without subsequent treatment. The GelMA@KGN group and the GelMA@KGN-BMSC group were administered a single intra-articular injection of GelMA@KGN microspheres and GelMA@KGN-BMSC microspheres, respectively, following the induction of OA.

#### 2.5.2 Establishment of TMJOA model

Based on previously established methodologies, a rabbit model of TMJOA was established via a single intra-articular injection of monosodium iodoacetate (MIA). Following anesthesia with 3% pentobarbital sodium (30 mg/kg) administered through the auricular vein, the animal was positioned supine and immobilized. The temporomandibular joint space was identified by palpation anterior to the bilateral tragus. The local hair was shaved, and the area was disinfected using povidone-iodine. Subsequently, 50 μL of MIA solution at a concentration of 10 mg/mL was injected into the upper joint cavity of both temporomandibular joints using arthrocentesis. The puncture was performed with care to prevent damage to surrounding joint tissues. After the injection, the mandible was gently moved several times to facilitate even distribution of the solution. The control group received an injection of 50 μL sterile normal saline. Following the injection, all rabbits received subcutaneous administrations of carprofen (4 mg/kg/day) for analgesia over a period of three days post-surgery to mitigate acute pain resulting from the surgical procedure and to promote normal feeding behavior. Disease progression was primarily assessed by behavioral observations and histological analysis. Within three days post-injection, all treated animals exhibited characteristic TMJOA-related behaviors on the operated side, including decreased chewing activity and prolonged feeding duration. Histological analysis demonstrated marked cartilage structural damage and loss after four weeks (**Supplementary Figure S1**).

#### 2.5.3 Intra-articular injection therapy

Four weeks following the completion of the MIA injection modeling, treatments were administered in accordance with the designated group assignments. GelMA@KGN microspheres and GelMA@KGN-BMSC microspheres were each suspended in sterile PBS at a concentration of 5 × 10^6^ particles/mL. A volume of 100 μL was injected into the joint cavity of one side of each animal, resulting in a total volume of 200 μL for both sides. Subsequent to the injection, the TMJ was gently mobilized to facilitate uniform distribution of the microspheres within the joint cavity. The pain management plan will continue as previously established. Postoperative monitoring included observation of the animals’ feeding behavior and general health status. All animals maintained under standard conditions until the designated sampling time points.

#### 2.5.4 Sample collection and processing

All experimental animals were euthanized and sampled at 4 and 8 weeks post-treatment. Euthanasia was conducted through the rapid intravenous administration of an overdose of pentobarbital sodium (120 mg/kg) via the auricular vein. Death was confirmed by the absence of respiration and cardiac activity for a minimum duration of three minutes. The bilateral temporomandibular joints were carefully isolated, with surrounding soft tissues meticulously removed, and the condyles were excised. The condylar tissues were fixed in 4% paraformaldehyde at 4 °C for 72 hours. Subsequently, the tissues were rinsed three times with PBS and transferred to a 10% EDTA solution for decalcification, which was conducted on a shaker at 37 °C. The decalcification solution was refreshed every three days, and the process continued for approximately four weeks. Completion of decalcification was confirmed by the absence of resistance to needle puncture.

### 2.6 Evaluation of therapeutic outcomes of GelMA@KGN-BMSC *in vivo*

#### 2.6.1 Histology, immunohistochemical and immunofluorescence analyses

Following decalcification, the samples were subjected to graded ethanol dehydration, xylene clearing, and paraffin embedding. Continuous sagittal sections with 4 μm thick were prepared, mounted on slides and baked overnight at 60 °C. The sections were stained with hematoxylin and eosin (HE) to evaluate overall morphology, Safranin O-Fast Green (SO-FG) and masson’s trichrome to assess collagen deposition, and COL-2 immunohistochemistry to examine the cartilage matrix. Cartilage degeneration was assessed in a blinded manner using the Mankin scoring system. For immunofluorescence staining, sections were deparaffinized, treated with 3% hydrogen peroxide for 10 minutes, permeabilized with 0.1% Triton X-100 for 30 minutes, and blocked with 5% BSA for an hour. Subsequently, the sections were incubated overnight at 4 °C with primary antibodies targeting ACAN, MMP13, or iNOS. Thereafter, the sections were incubated in the dark for 1 hour with goat anti-rabbit secondary antibodies, and nuclei were counterstained with DAPI. Images were acquired using a panoramic scanner or fluorescence microscope. Using ImageJ software at 100× magnification, three random fields per section were selected to quantify the percentage area positive for COL-2, the average fluorescence intensity of ACAN, and the percentage of cells positive for MMP13 or iNOS.

#### 2.6.2 Detection of cartilage and inflammation-related gene expression

As previously described, total RNA was extracted utilizing an RNA extraction kit, followed by reverse transcription into cDNA using a reverse transcription kit. The primer sequences employed in this study are provided in **Table 1**. Quantitative analysis of the expression levels of osteoarthritis-related marker genes in chondrocytes was conducted using qRT-PCR, focusing primarily on COL2A1, ACAN, MMP13, iNOS, and Arg-1. All results were normalized with respect to the measurements obtained on 4 weeks in Blank group.

### 2.7 Statistical analysis

All experimental data are expressed as mean ± standard deviation (mean ± SD). Prior to analysis, data were assessed for normality and homogeneity of variance. Statistical analyses were conducted using GraphPad Prism version 8.0. One-way analysis of variance (ANOVA) was employed for comparisons among multiple groups, while independent t-tests were used for comparisons between two groups. A p-value less than 0.05 was considered indicative of statistical significance.

## 3 Results

### 3.1 Material characterization of GelMA@KGN-BMSC microspheres

Under optical microscopy, the three groups of microspheres demonstrated uniform sizes (**Figure 1A**). Both the GelMA microspheres and GelMA@KGN microspheres exhibited regular, circular morphologies. BMSCs encapsulated within the GelMA@KGN-BMSC microspheres were distinctly observed, confirming the successful fabrication of the experimental group microspheres. SEM analysis revealed that the GelMA microspheres retained their spherical morphology following freeze-drying, with clearly defined surface material structures (**Figure 1B**).Particle size distribution analysis indicated that the average diameter of the microspheres was (106.36 ± 7.62), (103.47 ± 6.53), and (104.82 ± 8.21) μm, with a narrow size distribution, demonstrating that the microspheres produced via microfluidic control possessed high uniformity (**Figure 1C**). The average pore size of GelMA microspheres was (20.65 ± 4.30) μm, that of GelMA@KGN microspheres was (21.30 ± 4.61) μm, and that of GelMA@KGN-BMSC microspheres was (19.97 ± 3.77) μm. No statistically significant differences were observed among the groups, indicating that the incorporation of KGN and the encapsulation of BMSCs did not significantly affect the pore structure of the GelMA microspheres (**Figure 1D**).

**Figure 1:**
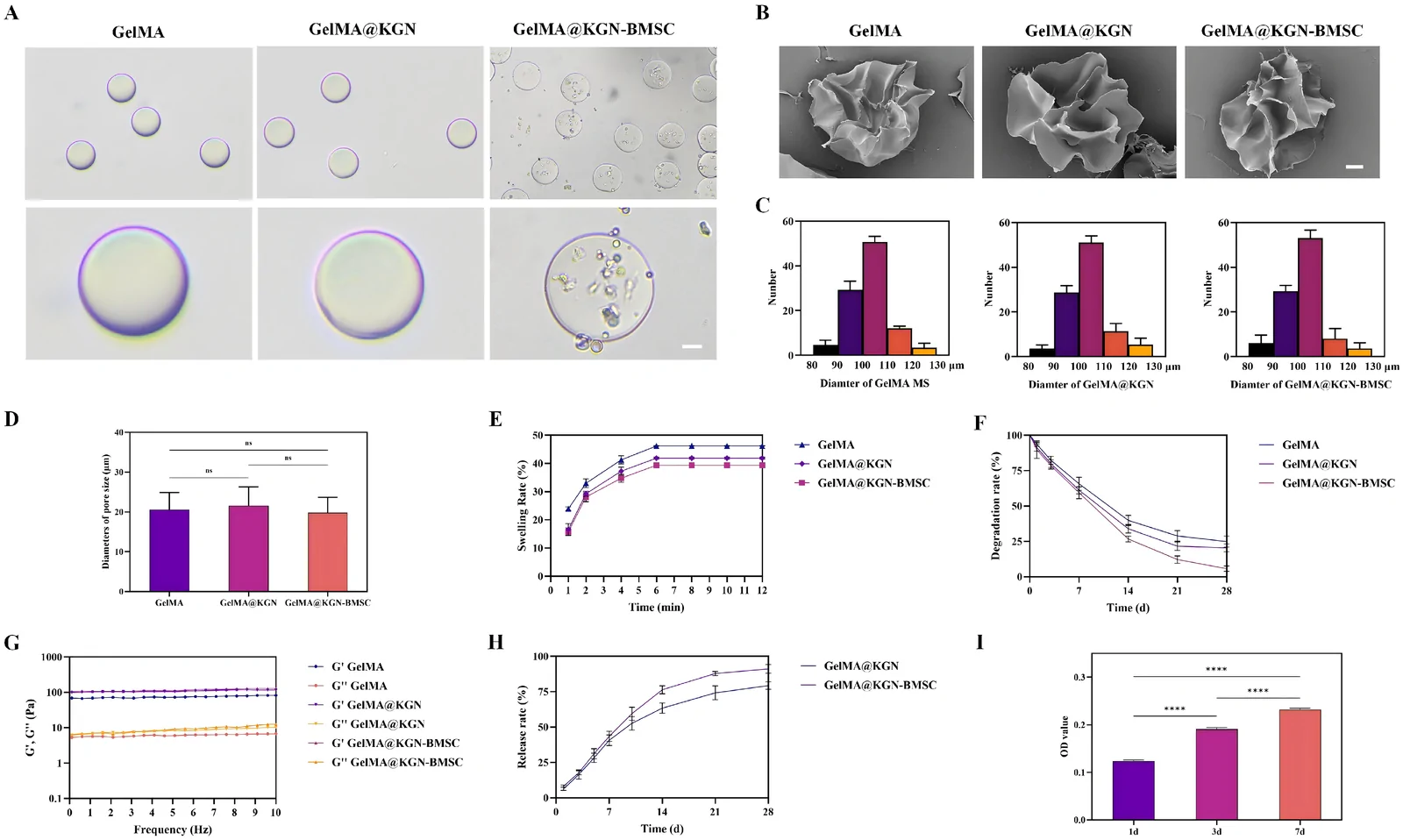
Characterization of GelMA@KGN-BMSC microspheres. **(A)** Morphological characterization of the three microsphere groups was conducted using optical microscopy, with a scale bar of 20 μm. **(B)** Surface morphology of freeze-dried GelMA microspheres was examined via SEM, with a scale bar of 10 μm. **(C)** Particle size distribution analysis of the three microsphere groups. **(D)** Diameters of pore size of three microsphere groups. **(E)** Swelling behavior of the three microsphere groups in PBS, reaching equilibrium at 6 hours. **(F)** Degradation profiles of the three microsphere groups in PBS containing 1 U/mL type I collagenase over a 28-day period. **(G)** Rheological assessment displaying the storage modulus (G’) and loss modulus (G’’) of the three microsphere groups across a frequency range of 0.1–10 Hz, with G’ significantly exceeding G’’. **(H)** Cumulative release profiles of KGN from GelMA@KGN and GelMA@KGN-BMSC microspheres over 28 days. **(I)** CCK-8 assay assessing the proliferative activity of BMSCs in GelMA@KGN-BMSC microspheres. Data are expressed as mean ± SD, with n = 3. Statistical significance is indicated as follows: *P < 0.05, **P < 0.01, ***P < 0.001, ****P < 0.0001.

Following rehydration in PBS, the swelling rates of the three groups of lyophilized microspheres increased over time, reaching equilibrium at approximately 6 hours. This result demonstrating that the microspheres possess substantial water absorption capacity, which is advantageous for maintaining their three-dimensional structure (**Figure 1E**). In PBS containing 1 U/mL type I collagenase, all three microsphere groups exhibited time-dependent degradation. The degradation rate was faster during the initial 14 days, followed by a gradual deceleration. By day 28, the mass retention rates were (24.83 ± 3.20)% for GelMA microspheres, (20.43 ± 2.29)% for GelMA@KGN microspheres, and (5.70 ± 1.59)% for GelMA@KGN-BMSC microspheres (**Figure 1F**). Rheological analysis revealed that the storage modulus (G’) of all three microsphere groups was significantly greater than the loss modulus (G’’) across the frequency range of 0.1–10 Hz, indicating the presence of a stable elastic gel network structure (**Figure 1G**). Collectively, these findings indicate that the incorporation of KGN and BMSCs did not significantly alter the material properties of the GelMA microspheres. (40.73 ± 3.07)% for GelMA@KGN and (43.43 ± 2.97)% for GelMA@KGN-BMSC of the drug was released within the first 7 days. After which the release rate stabilized, resulting in a cumulative release of (79.30 ± 2.18)% for GelMA@KGN and (91.13 ± 2.43)% for GelMA@KGN-BMSC by day 30 (**Figure 1H**). The release curve fits the Higuchi model, suggesting that the release mechanism is predominantly governed by diffusion.

In addition, the optical density (OD) values of BMSCs encapsulated within the microspheres exhibited a time-dependent increase over the culture period (**Figure 1I**). Specifically, the OD values measured on days 3 and 7 were significantly higher than those on day 1, indicating sustained metabolic activity and continued proliferation of BMSCs within the microspheres. These findings demonstrate that the porous architecture of the GelMA microspheres facilitates adequate nutrient exchange and oxygen diffusion, thereby supporting long-term cell viability and proliferation.

### 3.2 Biocompatibility analysis of GelMA@KGN-BMSC microspheres

Regarding cell morphology, phalloidin staining, Live/dead staining, and bright-field microscopy images corroborated chondrocytes adhered effectively to the surfaces of all three microsphere types and exhibited adequate spreading (**Figures 2A–C**). Regarding the influence of microspheres on cell viability, Live/Dead staining results demonstrated that after seven days of co-culture with microspheres, all groups exhibited a predominance of green-stained viable cells, with only occasional red-stained dead cells observed (**Figure 2D**). Quantitative image analysis revealed that the cell survival rate exceeded 85% across all groups (**Figure 2E**). Notably, chondrocytes treated with GelMA@KGN-BMSC microspheres displayed marked proliferation. Consistent with these observations, CCK-8 assays indicated a time-dependent increase in cell proliferation over the seven-day co-culture period with GelMA, GelMA@KGN, and GelMA@KGN-BMSC microspheres. Furthermore, significant differences in proliferation were observed at each time point between the GelMA@KGN and GelMA@KGN-BMSC groups compared to the other groups, with the GelMA@KGN-BMSC group exhibiting the most pronounced effect. These findings suggest that the microspheres and their constituent components are non-cytotoxic and that GelMA@KGN and GelMA@KGN-BMSC microspheres substantially enhance cell proliferation (**Figure 2F**).

**Figure 2:**
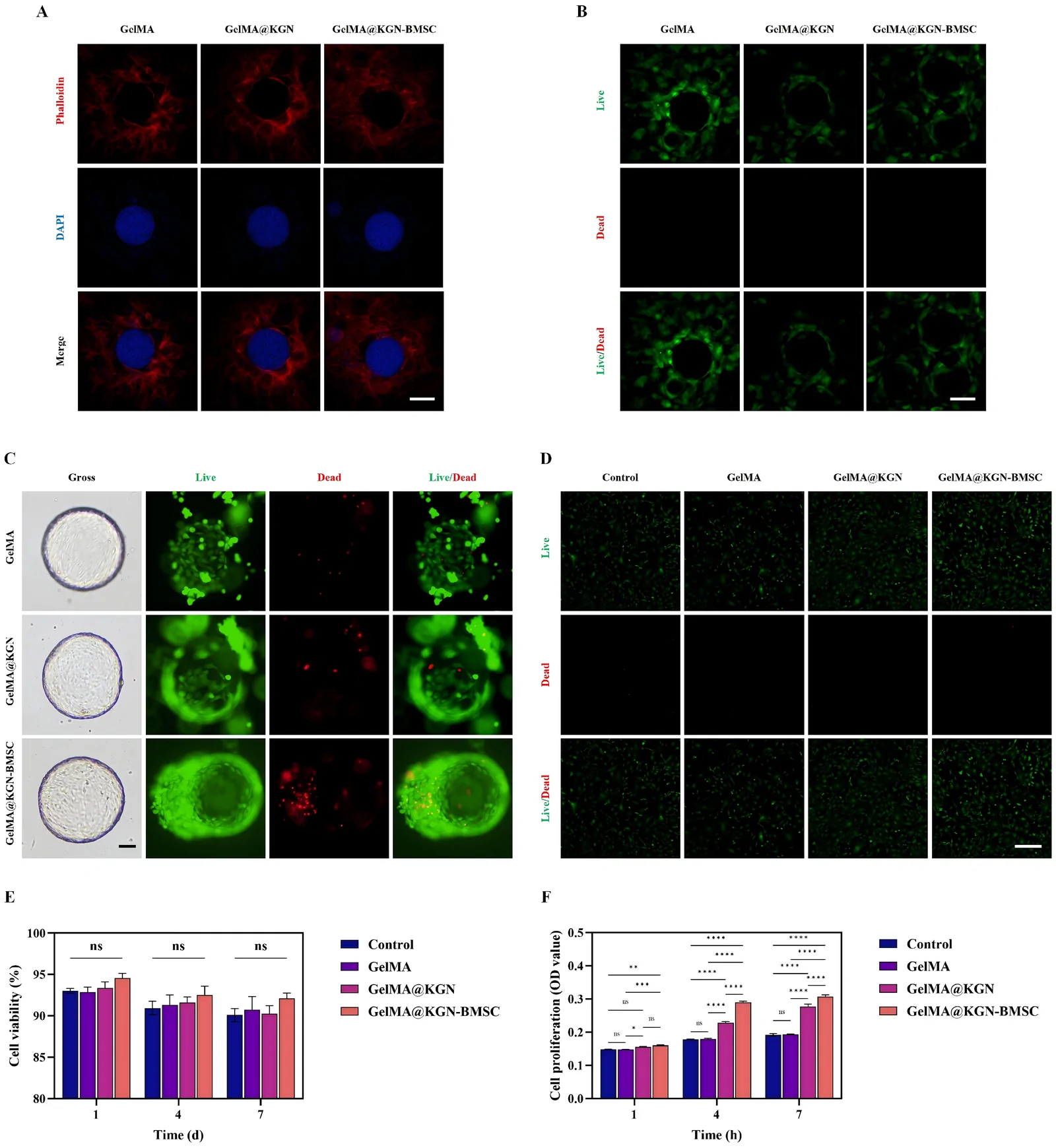
*In vitro* biocompatibility assessment of GelMA@KGN-BMSC microspheres. **(A)** Phalloidin staining was employed to examine the adhesion and spreading of chondrocytes on the surfaces of microspheres across three groups. Scale bar = 5 μm. **(B)** Live/Dead staining was performed to evaluate the viability of chondrocytes on the surfaces of microspheres across three groups. Scale bar = 5 μm. **(C)** Cells were observed to adhere to and spread on microspheres, as evidenced by bright-field and confocal microscopy. Scale bar = 20 μm. **(D)** Representative images of Live/Dead staining of chondrocytes following a 7-day co-culture with microspheres from three distinct groups are presented. Scale bar = 100 μm. **(E)** Quantitative analysis of cell survival rates was conducted for each group. **(F)** CCK-8 assay assessing the proliferative activity of chondrocytes following co-culture with microspheres from three distinct groups at 1, 4, and 7 days. Data are expressed as mean ± SD, with a sample size of n=3. Statistical significance is indicated as follows: *P < 0.05, **P < 0.01, ***P < 0.001, ****P < 0.0001.

### 3.3 Evaluation of cartilage related effects of GelMA@KGN-BMSC microspheres *in vitro*

In the gene expression analysis of chondrocytes *in vitro*, RT-qPCR results demonstrated that after 1 day of co-culture, the mRNA expression levels of cartilage marker genes COL2A1, ACAN, and SOX9 in both the GelMA@KGN-BMSC and GelMA@KGN groups were significantly elevated compared to those in the IL-1β group. Meanwhile, the expression levels of MMP13, ADAMTS5, TNF-α, and iNOS were significantly reduced relative to the IL-1β group (**Figures 3A–G**). No significant differences were observed in the expression levels of these genes between the GelMA@KGN-BMSC and GelMA@KGN groups at this time point. However, after 3 days of co-culture, these results changed markedly. Specifically, the mRNA expression levels of COL2A1, ACAN, and SOX9 in the GelMA@KGN-BMSC group were significantly higher than those in the GelMA@KGN group, while the expression levels of MMP13, ADAMTS5, TNF-α, and iNOS were significantly lower than those in the GelMA@KGN group.

**Figure 3:**
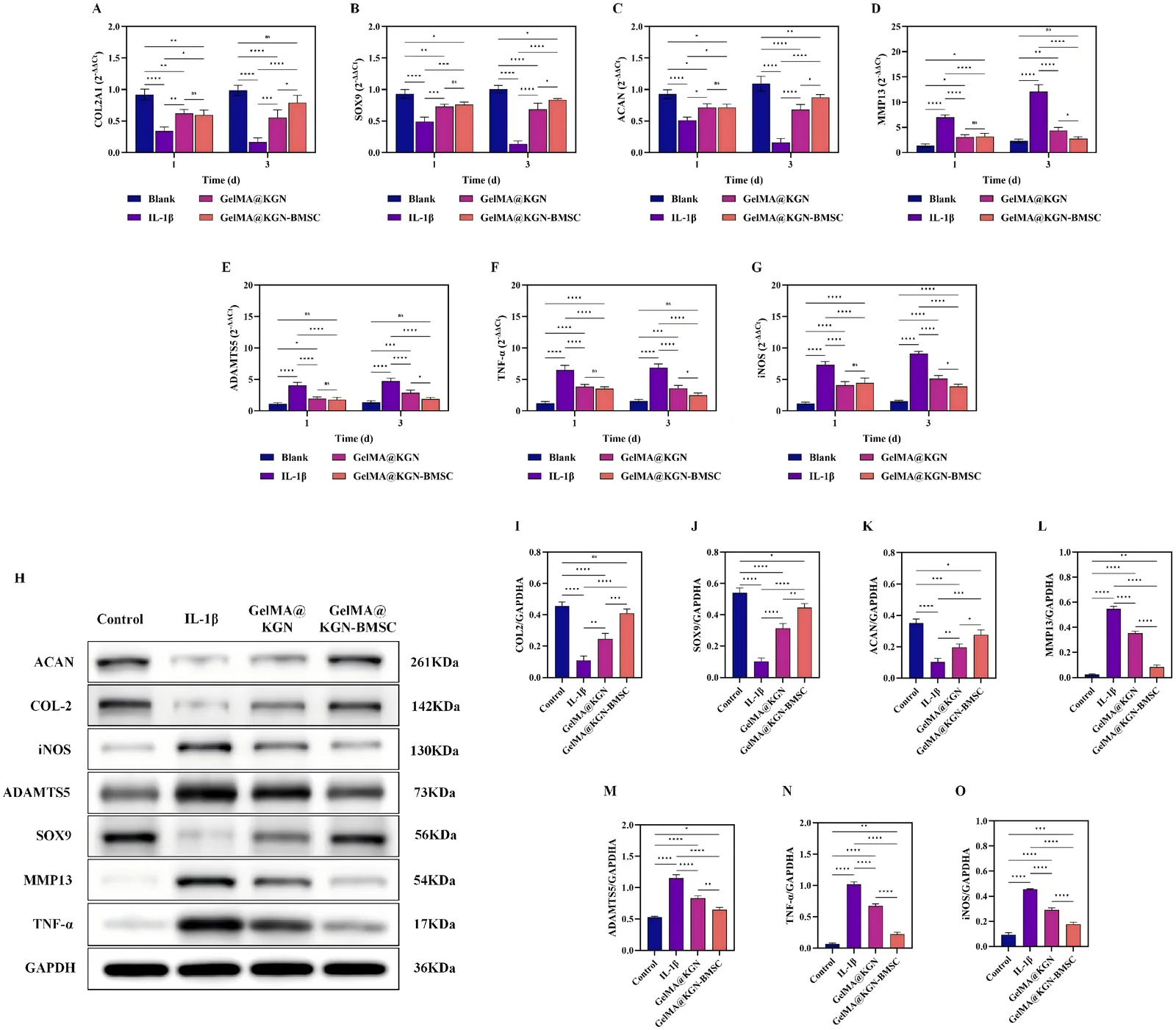
GelMA@KGN-BMSC microspheres enhance extracellular matrix synthesis by chondrocytes and inhibit its degradation *in vitro*. **(A–G)** RT-qPCR analysis of mRNA expression levels of COL2A1 **(A)**, SOX9 **(B)**, ACAN **(C)**, MMP13 **(D)**, ADAMTS5 **(E)**, TNF-α **(F)**, and iNOS **(G)** in chondrocytes following co-culture with three distinct groups of microspheres for 1 and 3 days. **(H)** Western blot analysis depicting protein expression of COL2A1, SOX9, ACAN, MMP13, ADAMTS5, TNF-α, and iNOS in chondrocytes from each group after 3 days of co-culture. **(I–O)** Quantitative assessment of the grayscale intensities corresponding to each protein band. Data are expressed as mean ± SD, with n = 3. Statistical significance is indicated as follows: *P < 0.05, **P < 0.01, ***P< 0.001, ****P< 0.0001.

Besides, total protein was extracted from chondrocytes in each group to assess the expression levels of chondrogenic differentiation markers (COL2A1, ACAN, SOX9), the matrix degradation marker (MMP13) and inflammation-related markers (ADAMTS5, TNF-α, iNOS) after 3 days of co-culture. As illustrated in **Figure 3H**, the protein expression levels of COL2A1, ACAN, and SOX9 in the GelMA@KGN-BMSC group were significantly elevated compared to those in the IL-1β group and the GelMA@KGN group. Conversely, the expression level of MMP13, ADAMTS5, TNF-α, and iNOS in the GelMA@KGN-BMSC group was significantly reduced relative to the other two groups (**Figures 3I–O**). Grayscale value analysis further confirmed this finding, aligning with the previously mentioned RT-qPCR results.

The results presented above demonstrate that GelMA@KGN-BMSC microspheres significantly enhance the synthesis of cartilage extracellular matrix and inhibit its degradation through a synergistic mechanism, exhibiting superior efficacy compared to GelMA@KGN microspheres.

### 3.4 Evaluation of anti-inflammatory function of GelMA@KGN-BMSC microspheres *in vitro*

After co-culturing RAW 264.7 macrophages with three groups of microspheres for 24 hours, the expression levels of iNOS, TNF-α, IL-1β, Arg-1, CD206, and IL-10 were assessed via immunofluorescence staining (**Figures 4A–F**). The results demonstrated that, in the GelMA@KGN-BMSC and GelMA@KGN microsphere group, the fluorescence intensities of iNOS, TNF-α, and IL-1β were markedly decreased, while those of Arg-1, CD206, and IL-10 were significantly elevated, suggesting that macrophages displayed the most pronounced polarization toward the M2 anti-inflammatory phenotype.

**Figure 4:**
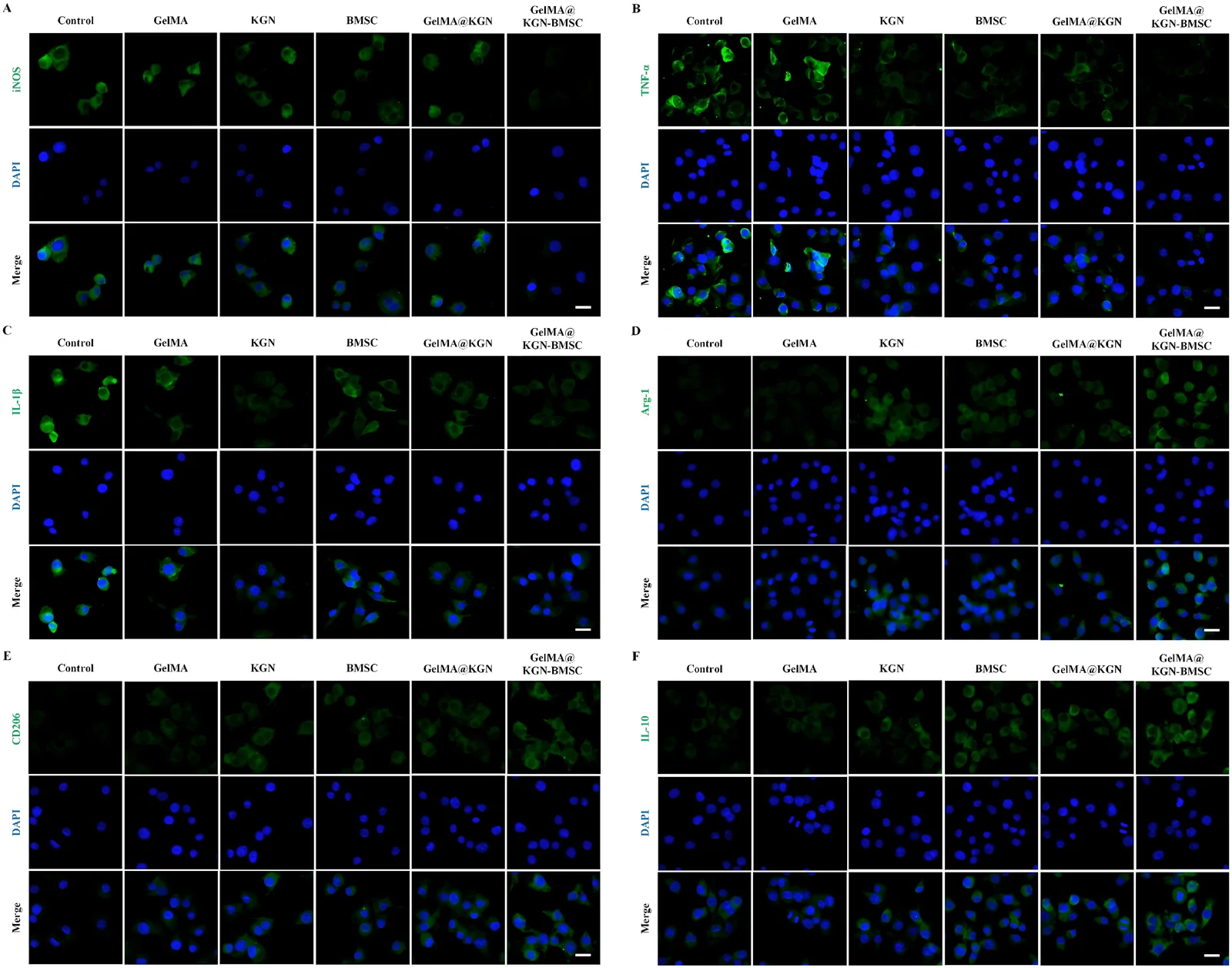
GelMA@KGN-BMSC microspheres promote macrophage polarization toward the M2 phenotype and modulate the secretion of inflammatory factors *in vitro*. Immunofluorescence staining for the expression of iNOS **(A)**, TNF-α **(B)**, IL-1β **(C)**, Arg-1 **(D)**, CD206 **(E)**, and IL-10 **(F)** in RAW 264.7 macrophages following 24 hours of co-culture with three distinct groups of microspheres. Scale bar = 20 μm.

After 48 hours of co-culture, the cell supernatant was collected to measure the concentrations of inflammatory factors (**Figures 5A–F**). Compared to the control group, the GelMA@KGN-BMSC microsphere group exhibited a significant reduction in the levels of iNOS, TNF-α, and IL-1β, alongside a significant increase in the concentrations of Arg-1, CD206, and IL-10. Furthermore, a statistically significant difference was observed between the GelMA@KGN microsphere group and the GelMA@KGN-BMSC microsphere group. Additionally, total RNA was extracted from macrophages to assess the mRNA expression of M1 and M2 marker genes (**Figures 5G–L**). The data demonstrated that the mRNA expression levels of iNOS, TNF-α, and IL-1β in the GelMA@KGN microsphere group were significantly lower than those in the control group, whereas the expression levels of Arg-1, CD206, and IL-10 were significantly elevated. These findings were consistent with the ELISA results, revealing significant differences between the GelMA@KGN and GelMA@KGN-BMSC microsphere groups.

**Figure 5:**
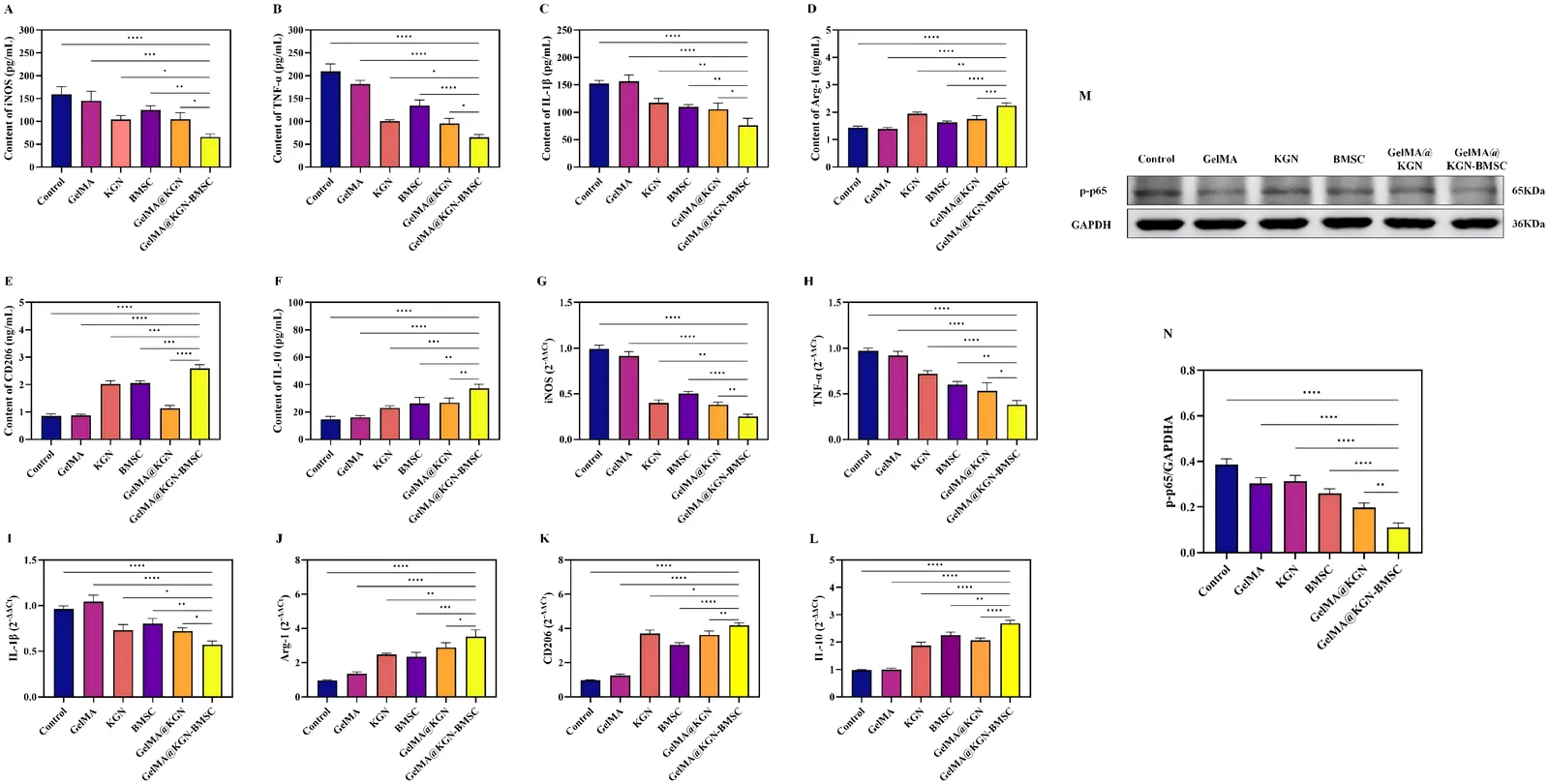
GelMA@KGN-BMSC microspheres promote macrophage polarization toward the M2 phenotype and modulate the secretion of inflammatory factors *in vitro*. A-F show ELISA measurements of protein concentrations of iNOS **(A)**, TNF-α **(B)**, IL-1β **(C)**, Arg-1 **(D)**, CD206 **(E)**, and IL-10 **(F)** in the culture supernatant after 48 hours of co-culture. G–L present RT-qPCR analysis of mRNA expression levels of iNOS **(G)**, TNF-α **(H)**, IL-1β **(I)**, Arg-1 **(J)**, CD206 **(K)**, and IL-10 **(L)** in macrophages after 48 hours of co-culture. **(M, N)** GelMA@KGN-BMSC microspheres mediates anti-inflammatory effects by suppressing the phosphorylation activation of the NF-κB signaling pathway. Data are expressed as mean ± SD, with n = 3. Statistical significance is indicated as follows: *P < 0.05, **P < 0.01, ***P< 0.001, ****P< 0.0001.

Western blot analysis demonstrated that, compared to the control group, the expression levels of p-p65 protein were significantly reduced in both the GelMA@KGN group and the GelMA@KGN-BMSC group (**Figures 5M, N**). Notably, the GelMA@KGN-BMSC group exhibited a more pronounced inhibitory effect, with a statistically significant difference relative to the GelMA@KGN group. These findings suggest that the GelMA@KGN-BMSC microspheres mediates anti-inflammatory effects by suppressing the phosphorylation activation of the NF-κB signaling pathway.

Collectively, these results suggest that microspheres loaded with KGN effectively induce macrophage polarization toward the M2 anti-inflammatory phenotype and suppress the secretion of pro-inflammatory factors. Moreover, BMSCs appear to play a crucial role in modulating inflammation *in vitro* via paracrine mechanisms.

### 3.5 Evaluation of therapeutic effect of TMJOA model with GelMA@KGN-BMSC microspheres *in vivo*

According to the previous description, condyle process samples were collected at 4 and 8 weeks post-treatment to facilitate the evaluation of therapeutic outcomes in TMJOA. Histological analyses were performed to examine pathological changes in the articular cartilage and subchondral bone. HE staining revealed that the control group maintained a normal physiological condylar structure, characterized by an intact cartilage architecture and well-defined lacuna. In contrast, the OA group demonstrated hallmark pathological features of TMJOA, including disorganized cartilage layers, a roughened surface, and pronounced subchondral bone damage (**Figure 6A**). The GelMA@KGN group exhibited markedly attenuated cartilage damage relative to the OA group. Notably, the GelMA@KGN-BMSC group showed histological characteristics most closely resembling those of the control group, indicating minimal pathological deviation.

**Figure 6:**
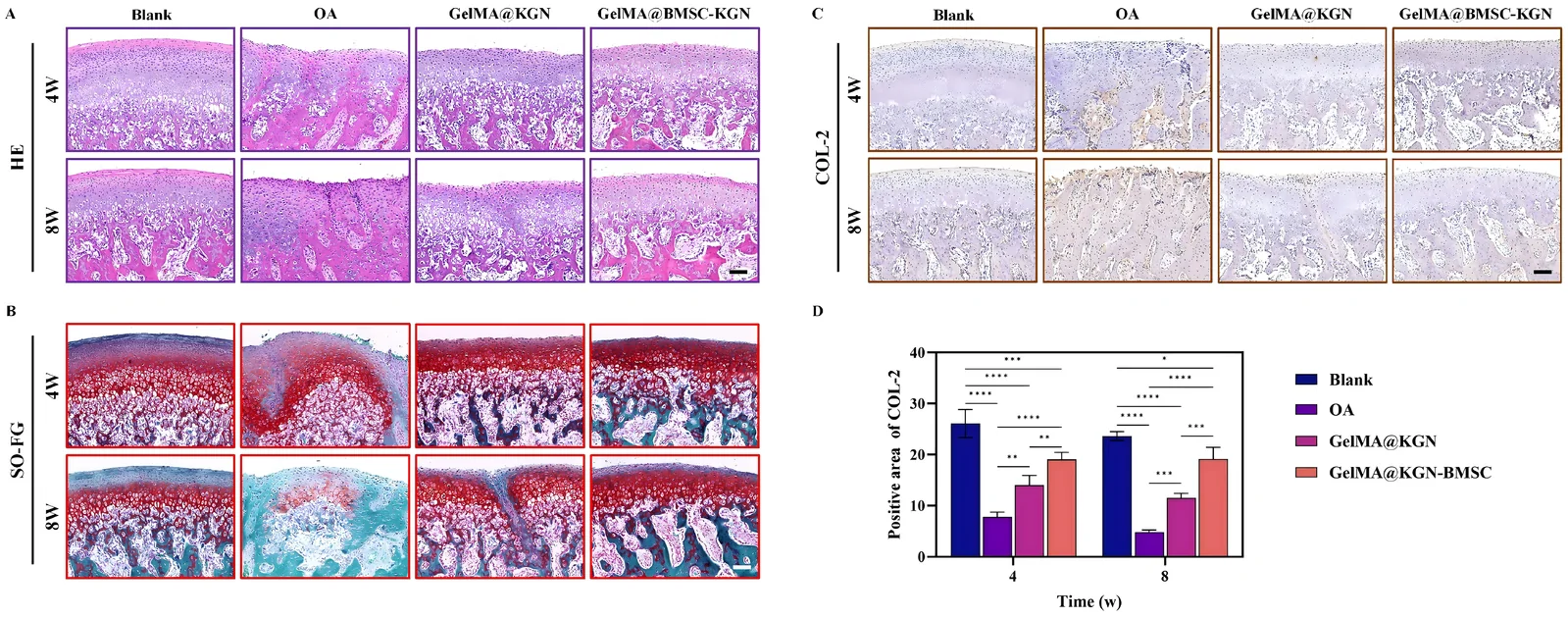
Enhancement of cartilage tissue architecture in a TMJOA model using GelMA@KGN-BMSC microspheres. **(A)** HE staining of condyles from each group at 4 and 8 weeks post-treatment; scale bar = 100 μm. **(B)** SO-FG staining to evaluate GAG deposition in cartilage; scale bar = 100 μm. **(C)** Immunohistochemical staining for COL-2; scale bar = 100 μm. **(D)** Quantitative analysis of the COL-2 positive area. Data are expressed as mean ± SD, with n = 3. Statistical significance is indicated as follows: *P < 0.05, **P < 0.01, ***P< 0.001, ****P< 0.0001.

To evaluate the deposition of glycosaminoglycans (GAG) within the cartilage, SO-FG staining was conducted (**Figure 6B**). The findings indicated that although the GelMA@KGN group exhibited improved outcomes relative to the OA group, the SO-positive matrix in the cartilage was markedly reduced and unevenly distributed, showing a significant decrease compared to the control group. In contrast, the GelMA@KGN-BMSC group demonstrated a uniform distribution and abundant presence of the SO-positive matrix, suggesting enhanced GAG synthesis. Furthermore, this group preserved normal cartilage thickness, indicating a significant protective effect against cartilage matrix degradation.

COL-2 and ACAN as essential components of cartilage formation, were evaluated respectively through immunohistochemistry and immunofluorescence staining. Compared to the control group, the expression levels of COL-2 and ACAN were significantly reduced in the OA group (**Figures 6C, D**, **7A, E**). Following treatment with GelMA@KGN and GelMA@KGN-BMSC microspheres, the expression of COL-2 and ACAN was substantially increased, with the GelMA@KGN-BMSC group exhibiting the most pronounced enhancement. These findings suggest that GelMA@KGN-BMSC treatment effectively promotes cartilage matrix regeneration and anabolic activity.

**Figure 7:**
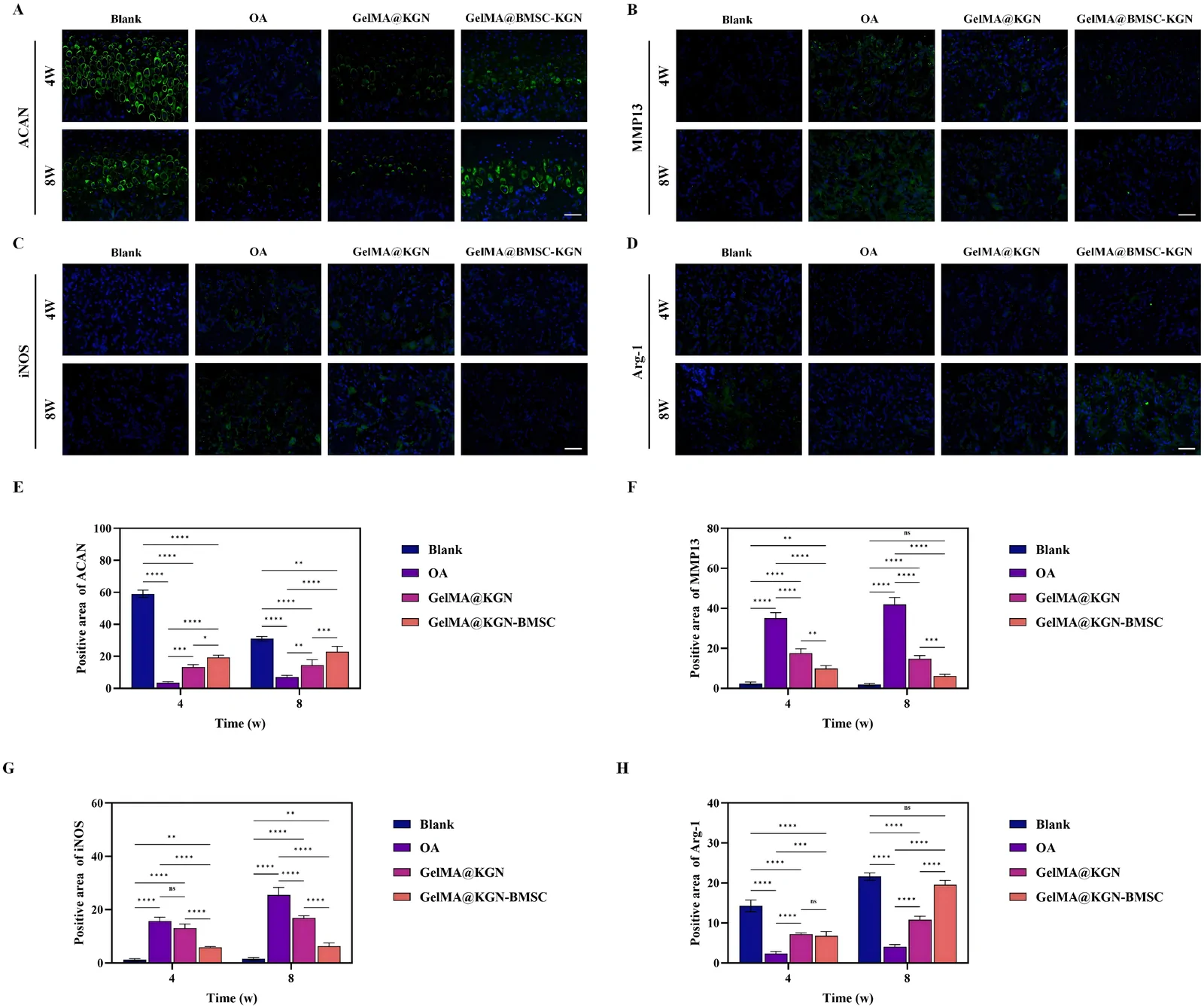
GelMA@KGN-BMSC microspheres enhance the synthesis of cartilage matrix and suppress inflammatory responses. **(A–D)** Representative immunofluorescence images depicting ACAN **(A)**, MMP13 **(B)**, iNOS **(C)**, and Arg-1 **(D)** expression in condylar tissues from each experimental group at 8 weeks post-treatment. Scale bar = 50 μm. **(E–H)** Quantitative analyses of the average fluorescence intensities corresponding to ACAN **(E)**, MMP13 **(F)**, iNOS **(G)**, and Arg-1 **(H)**. Data are expressed as mean ± SD, with n = 3. Statistical significance is indicated as follows: *P < 0.05, **P < 0.01, ***P< 0.001, ****P< 0.0001.

MMP13 plays a critical role in the degradation of the cartilage matrix. In this study, immunofluorescence staining was employed to detect MMP13 and to investigate the effect of GelMA@KGN-BMSC microspheres on mitigating cartilage inflammation (**Figures 7B, F**). The findings demonstrated a significant increase in MMP13 expression in the OA group, whereas treatment with GelMA@KGN-BMSC microspheres markedly reduced MMP13 expression, indicating its potential to inhibit cartilage catabolism. Additionally, immunofluorescence staining was utilized to assess the expression levels of iNOS and Arg1 to evaluate the anti-inflammatory effects of GelMA@KGN-BMSC *in vivo*. The results revealed a significant upregulation of Arg1 and a pronounced downregulation of iNOS in the GelMA@KGN-BMSC group (**Figures 7C, D, G**, **6H**). At the same time, the qRT-PCR results further corroborated the staining findings (**Figure 8**). These findings are consistent with the results obtained from *in vitro* cellular assays.

**Figure 8:**
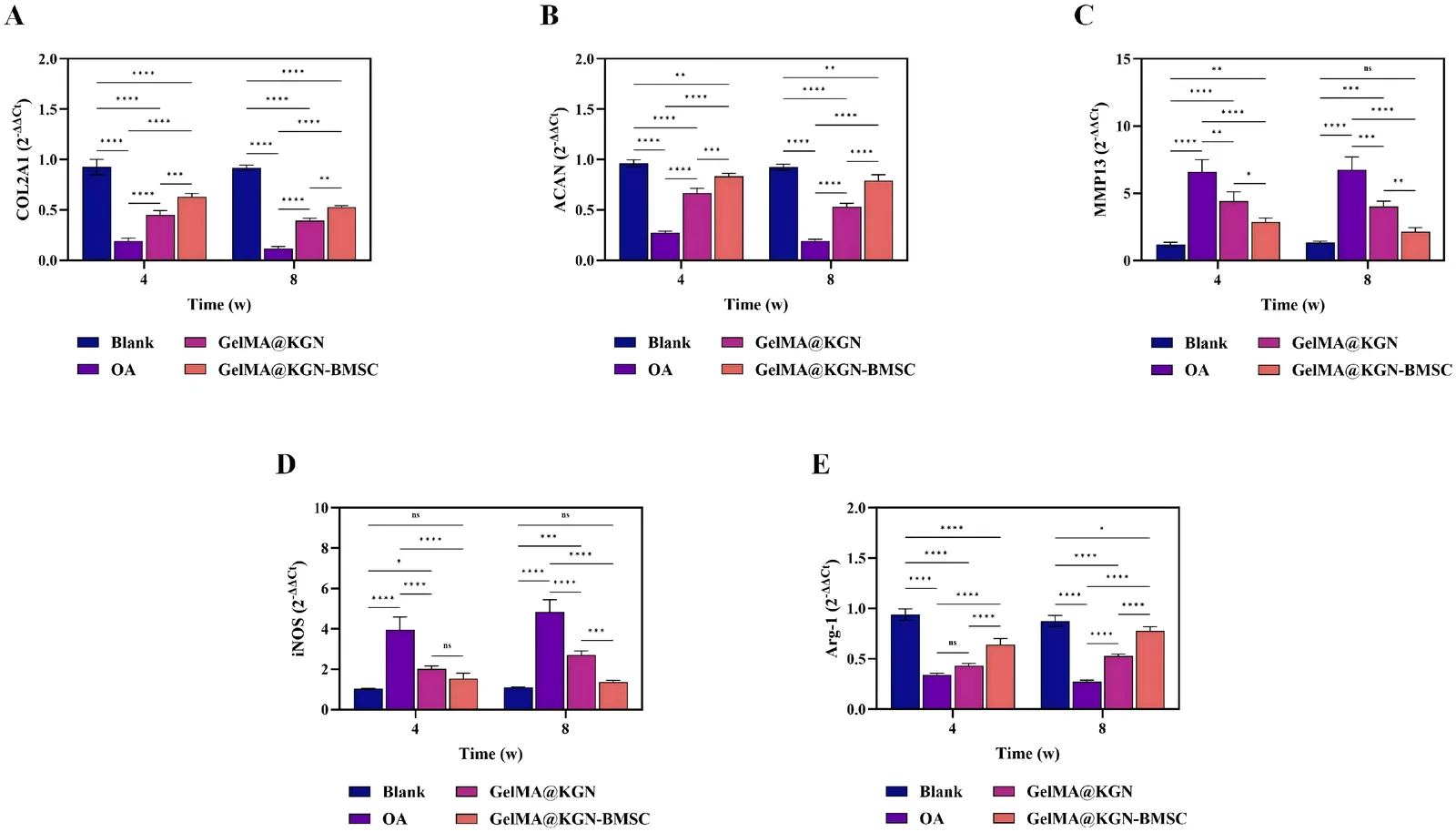
Regulation of Cartilage-Related Gene Expression in GelMA@KGN-BMSC Microspheres. RT-qPCR analysis was performed to assess the mRNA expression levels of COL2A1 **(A)**, ACAN **(B)**, MMP13 **(C)**, iNOS **(D)**, and Arg-1 **(E)** in condylar tissues from each group eight weeks post-treatment. Data are expressed as mean ± SD, with n = 3. Statistical significance is indicated as follows: *P < 0.05, **P < 0.01, ***P< 0.001, ****P< 0.0001.

## 4 Discussion

This study successfully developed a porous GelMA microsphere system loaded with rabbit BMSCs and KGN, designated GelMA@KGN-BMSC, and evaluated its biocompatibility, chondrogenic induction capacity, and therapeutic efficacy for TMJOA through both *in vitro* assays and an *in vivo* rabbit TMJOA model. The findings demonstrated that the microsphere system exhibits favorable physicochemical characteristics and biocompatibility. The porous architecture of the microspheres facilitates BMSCs adhesion, viability, and functional activity. Furthermore, the sustained release of KGN from the microspheres effectively promotes the differentiation of BMSCs into chondrocytes. Concurrently, the paracrine effects of BMSCs synergize with KGN to exert anti-inflammatory effects. Intra-articular administration of GelMA@KGN-BMSC microspheres significantly mitigated cartilage degeneration in the TMJOA model, enhanced cartilage matrix synthesis, and suppressed the expression of inflammatory mediators, with therapeutic outcomes substantially superior to those observed following treatment with GelMA@KGN microspheres alone.

An ideal intra-articular delivery carrier should exhibit excellent biocompatibility, an appropriate degradation rate, sufficient mechanical support, and a microenvironment conducive to cell adhesion and proliferation. GelMA hydrogels have been extensively utilized in cartilage tissue engineering due to their tunable physicochemical properties, photo-crosslinking capabilities, and superior biocompatibility. In the present study, GelMA microspheres prepared using microfluidic technology demonstrated uniform particle size and a porous architecture. SEM analysis revealed well-connected pores on both the surface and interior of the microspheres, facilitating nutrient exchange and the removal of cellular metabolic waste. Compared to dense microspheres, the porous structure offers BMSCs an increased adhesion area and a three-dimensional growth environment, which likely accounts for the observed enhanced cell spreading on the microsphere surface and high cell viability, as evidenced by Live/Dead staining and SEM observations. Furthermore, the swelling behavior and degradation profile of GelMA microspheres demonstrate their capacity to maintain structural integrity within the joint cavity for a minimum of four weeks, aligning with the *in vitro* release profile of KGN. The sustained release of KGN not only continuously promotes the chondrogenic differentiation of BMSCs but may also provide prolonged anti-inflammatory protection through activation of the NRF2 signaling pathway. In contrast, the direct administration of free KGN or free BMSCs is limited by rapid clearance, thereby impeding the achievement of comparable therapeutic outcomes. Consequently, GelMA porous microspheres serve as an effective co-delivery platform for both cells and drugs, addressing the dual challenges of low BMSC survival rates and the short half-life of KGN. Compared to chondrocytes, BMSCs offer several advantages as seed cells. Firstly, the procurement of BMSCs is less invasive and can be performed through minimally invasive techniques such as bone marrow aspiration, whereas harvesting chondrocytes typically necessitates the sacrifice of healthy articular cartilage tissue. Secondly, BMSCs exhibit a greater capacity for expansion *in vitro*, allowing for amplification to quantities sufficient to meet the demands of tissue engineering applications. In contrast, primary chondrocytes have limited proliferative potential *in vitro* and are susceptible to dedifferentiation, which hinders their large-scale acquisition. Thirdly, beyond their ability to differentiate directly into chondrocytes, BMSCs possess significant paracrine functions. BMSCs modulate the local microenvironment, inhibit apoptosis, and promote angiogenesis and tissue repair through the secretion of exosomes and various growth factors. These properties represent advantages not observed in terminally differentiated chondrocytes. Extensive research has demonstrated that BMSCs are more readily obtainable and possess a greater proliferative capacity in comparison to chondrocytes, rendering them more suitable as seed cells for tissue engineering approaches aimed at treating osteoarthritis. Furthermore, studies have revealed that BMSCs are capable of self-assembling into cartilage organoids within three-dimensional culture systems, exhibiting enhanced cell viability, increased proliferative potential, and significantly elevated expression of cartilage-specific markers. Additionally, the literature indicates that BMSCs can be expanded on a large scale *in vitro* without compromising their multipotent differentiation capabilities and provoke minimal immune rejection during allogeneic transplantation, thereby establishing their pivotal role in tissue engineering and regenerative repair applications. Moreover, the anti-inflammatory and immunomodulatory properties of BMSCs have been extensively validated. These cells modulate the inflammatory microenvironment of the joint cavity by promoting macrophage polarization toward the M2 anti-inflammatory phenotype, thus fostering conditions conducive to cartilage repair.

The principal innovation of this study is the co-encapsulation of KGN and BMSCs within GelMA microspheres, which facilitates a synergistic enhancement of drug-assisted cell therapy. *In vitro* experiments demonstrated that, following a 7 days co-culture of GelMA@KGN-BMSC microspheres with chondrocytes, the mRNA and protein expression levels of COL2A1, ACAN, and SOX9 were significantly elevated compared to those observed in the GelMA@KGN microsphere group and the GelMA microsphere group. Concurrently, the expression of MMP13 was markedly decreased. These findings suggest that although GelMA@KGN microspheres lacking BMSCs can moderately promote cartilage matrix synthesis, the presence of BMSCs substantially amplifies this effect. This enhancement is likely attributable to the differentiation of BMSCs into chondrocytes under the inductive influence of KGN within the microspheres. Additionally, paracrine factors secreted by BMSCs further stimulate the matrix synthesis activity of chondrocytes in the co-culture system, resulting in a dual mechanism of enhancement mediated by both cellular differentiation and paracrine signaling. Previous studies have demonstrated that KGN activates the NRF2 pathway and downregulates the expression of MMP13 and iNOS, thereby exerting a pronounced anti-inflammatory effect. In the present study, the GelMA@KGN-BMSC group significantly reduced the expression levels of iNOS and MMP13 both *in vivo* and *in vitro*, exhibiting superior efficacy compared to the GelMA@KGN microsphere group. Macrophage polarization assays revealed that RAW 264.7 cells co-cultured with GelMA@KGN-BMSC microspheres showed a significant upregulation of M2-type markers alongside a downregulation of M1-type markers. Concurrently, the secretion of anti-inflammatory cytokines increased, whereas pro-inflammatory cytokine levels decreased. These findings indicate that the immunomodulatory capacity of BMSCs synergizes with the anti-inflammatory properties of KGN to collectively mitigate the chronic inflammatory microenvironment within the TMJOA joint cavity. Consequently, the GelMA@KGN-BMSC microsphere system employed in this study not only directly suppresses inflammation by KGN but also indirectly modulates immune responses through BMSCs, thereby establishing a dual anti-inflammatory mechanism.

In the MIA induced rabbit TMJOA model, a single intra-articular injection of GelMA@KGN-BMSC microspheres administered at 4 and 8 weeks resulted in significant improvements in cartilage structure, as evidenced by histological evaluation. This was demonstrated by a marked increased area positive for COL-2. Conversely, the OA group and the GelMA@KGN microsphere group exhibited cartilage layer thinning, matrix fissure formation, even cartilage loss. Masson’s trichrome staining further confirmed that the GelMA@KGN-BMSC microsphere group displayed a well-organized collagen fiber arrangement and a significantly larger area of blue staining relative to the GelMA@KGN microsphere group, indicating effective remodeling of the subchondral bone and cartilage matrix collagen. Immunofluorescence analysis revealed enhanced ACAN expression in the GelMA@KGN-BMSC group, with a significantly lower percentage of MMP13 and iNOS positive cells compared to the GelMA@KGN microsphere group. The results presented above demonstrate that, although the GelMA@KGN microsphere group can partially mitigate cartilage degeneration relative to the OA group, it fails to achieve complete cartilage tissue regeneration and repair due to the absence of cellular components. In contrast, the GelMA@KGN-BMSC microspheres deliver exogenous BMSCs, which, under the directed induction of KGN, fulfill the dual objectives of cartilage structural restoration and matrix synthesis. Importantly, the therapeutic efficacy of the GelMA@KGN-BMSC group at 8 weeks surpassed that observed at 4 weeks, indicating that this microsphere system possesses sustained treatment capabilities. Furthermore, no significant adverse effects, such as joint cavity adhesion, infection, or ectopic migration of microspheres, were detected throughout the study, suggesting that the GelMA@KGN-BMSC microspheres exhibit favorable safety profiles.

In recent years, multiple research groups have investigated hydrogel microspheres loaded with KGN or BMSCs for the treatment of OA. A study developed gelatin microspheres loaded with KGN, and *in vitro* experiments demonstrated their efficacy in delivering KGN and promoting the chondrogenic differentiation of human mesenchymal stem cells. The expression levels of chondrogenic marker genes, including SOX9, ACAN, COMP, and COL2A1, were significantly upregulated (39). Yuan et al. developed an injectable hydrogel composed of chitosan and chondroitin sulfate encapsulating KGN-loaded PLGA microspheres, employing ultrasound to induce the controlled release of KGN. This system sustained drug concentration for a minimum of 28 days and enhanced COL-II synthesis in BMSCs (40). In addition, in the application of GelMA microspheres, Li X, et al. developed GelMA microspheres incorporating KGN and demonstrated their efficacy in inhibiting cartilage degradation in a knee OA model (41). Similarly, Yang Y, et al. encapsulated BMSCs within GelMA microspheres to repair cartilage defects, reporting favorable outcomes in cartilage regeneration (42). Furthermore, the GelMA@Lipo@KGN microspheres developed by Cui et al. demonstrated a joint retention duration exceeding five weeks in a surgically induced rat OA model (43). These microspheres effectively mitigated osteophyte formation and inhibited the degeneration of articular cartilage as well as alterations in subchondral bone. In recent research, GelMA hydrogel microspheres fabricated by microfluidic technology, to load predifferentiated BMSCs onto the microspheres, thereby constructing cartilage organoid precursors. This methodology demonstrated superior cartilage regeneration in a rat model of knee cartilage defects (44). Nevertheless, to date, no studies have reported the co-encapsulation of BMSCs and KGN within a single microsphere system for the treatment of TMJOA. This study represents the first integration of BMSCs therapy with KGN chemical induction on a GelMA microsphere platform, demonstrating their synergistic enhancement effects in a TMJOA model.

Despite the advancements achieved through the individual application of KGN microspheres or BMSCs microspheres in the studies mentioned above, there have been no reports on the co-loading of BMSCs and KGN within a single microsphere for the treatment of TMJOA. This study is the first to combine BMSCs therapy with KGN drug induction encapsulated within GelMA microspheres and to demonstrate their synergistic enhancement effect in a TMJOA model. Compared to prior research, the novelty of this investigation is reflected in the following aspects. Firstly, this study focuses on the TMJ, a distinct anatomical structure whose mechanical environment and pathological characteristics differ markedly from those of the knee joint. While the majority of prior research has centered on knee OA, the microenvironment of TMJOA exhibits unique features that warrant specific investigation. Secondly, by incorporating a GelMA@KGN microsphere control group, the study effectively differentiates the therapeutic effects of drug-only treatment from those of combined cell and drug therapy, demonstrating that the addition of BMSCs confers advantages beyond KGN treatment alone. Furthermore, the study systematically elucidates the synergistic mechanisms underlying these effects from multiple perspectives, including chondrogenic differentiation, anti-inflammatory responses, macrophage polarization, and modulation of the NF-κB signaling pathway. The findings reveal that the microsphere system mediates its anti-inflammatory and M2 macrophage polarization-promoting effects through inhibition of NF-κB phosphorylation at the molecular level. Furthermore, the GelMA material employed in this study exhibits excellent biocompatibility and degradability, thereby providing BMSCs with a microenvironment that closely mimics the natural cartilage ECM. In summary, this study proposes a novel minimally invasive therapeutic strategy that integrates cellular and pharmacological approaches for the treatment of TMJOA. The synergistic enhancement mechanism and targeted application to the TMJ constitute a significant advancement relative to existing research.

It is important to consider whether the anti-inflammatory properties of the microspheres might excessively suppress inflammation, thereby adversely affecting the quality of cartilage regeneration. Recent studies have demonstrated that moderate inflammatory signaling plays a critical role in initiating and sustaining tissue repair processes (45). However, the GelMA@KGN-BMSC employed in this study does not seek to completely inhibit inflammation. Instead, it facilitates macrophage polarization from the pro-inflammatory M1 phenotype to the anti-inflammatory M2 phenotype, thereby transforming chronic inflammation, which typically causes tissue damage, into a reparative inflammatory microenvironment. The paracrine activity of BMSCs endows GelMA@KGN-BMSC with a distinctive capacity to regulate the balance between inflammation and regeneration, dynamically modulating the secretion of anti-inflammatory and pro-repair signals in response to local inflammatory status. Furthermore, KGN not only suppresses inflammation but also promotes the chondrogenic differentiation of BMSCs, thereby further enhancing tissue regeneration. Consistently, our *in vivo* experiments confirmed that the GelMA@KGN-BMSC group exhibited the most intact cartilage architecture, the highest expression levels of COL-II and ACAN, and showed no adverse repair outcomes such as fibrocartilage formation or hypertrophic cartilage development. In summary, GelMA@KGN-BMSC mediates immune remodeling of the inflammatory microenvironment, establishing a balance between inflammation resolution and cartilage regeneration that supports high-quality tissue repair.

This study has several acknowledged limitations. First, although *in vitro* experiments demonstrated the favorable survival of BMSCs within GelMA microspheres, the long-term fate of BMSCs following *in vivo* injection was neither tracked nor observed. Future studies will involve labeling BMSCs via lentiviral transfection to enable dynamic monitoring using an animal live imaging system *in vivo*. Second, the observation period was limited to 8 weeks, leaving the long-term durability of cartilage repair uncertain. Third, while the release profile of KGN conformed to the Higuchi model, individual variability in release rates necessitates further optimization. Future research will investigate the potential of bilayer microsphere structures to achieve more precise, programmed release. Fourth, this study utilized rabbit auricular chondrocytes as target cells in *in vitro* experiments due to their ease of acquisition, robust proliferative capacity, and favorable chondrogenic potential in three-dimensional culture. Nevertheless, inherent differences exist between auricular cartilage and TMJ condylar cartilage in terms of histological phenotype and mechanical properties, constituting a methodological limitation of the present study. Future investigations will employ primary rabbit TMJ condylar chondrocytes for *in vitro* validation to more accurately assess the effects of GelMA@KGN-BMSC on chondrocytes derived from the relevant tissue. Fifth, a biomaterial control group consisting solely of GelMA microspheres was not established in this study. Therefore, the potential nonspecific effects of the GelMA matrix on the joint microenvironment cannot be entirely excluded. Although *in vitro* experiments have demonstrated that GelMA microspheres do not significantly influence chondrocyte proliferation, and GelMA has been extensively validated for its excellent *in vivo* biocompatibility, nonspecific effects may still occur in the *in vivo* environment that differ from those observed *in vitro*. Future studies will incorporate a control group with GelMA microspheres alone to assess the biomaterial’s impact on the repair outcomes of TMJOA. Sixth, in this study, free BMSCs and free KGN solution were not concurrently administered into the joint cavity. The lack of this control group limits our ability to directly compare and demonstrate the superior therapeutic efficacy of microsphere co-delivery relative to conventional intra-articular injection. Future experiments will include supplementary tests with single-material groups to further substantiate the benefits of microsphere co-delivery. Additionally, the animal model employed involved a single injection of MIA, which induces cartilage degeneration but does not replicate the gradual progression characteristic of human TMJOA. Subsequently, we will consider validating these findings using mechanical overload or spontaneous OA models.

In summary, the GelMA@KGN-BMSC microsphere system developed in this study enables localized delivery of BMSCs and sustained release of KGN through a single intra-articular injection. The GelMA@KGN-BMSC microspheres synergistically combine the chondrogenic induction and anti-inflammatory properties of KGN with the paracrine immunomodulatory functions of BMSCs, thereby effectively promoting cartilage regeneration, inhibiting matrix degradation, and attenuating inflammatory responses in the TMJOA model. Compared to treatment with GelMA@KGN microspheres alone, the incorporation of BMSCs significantly enhanced therapeutic efficacy, underscoring the fundamental advantage of this tissue engineering approach. This strategy facilitates functional repair through the use of living cells rather than solely providing symptomatic relief via pharmacological agents. Consequently, it represents a novel minimally invasive therapeutic option for TMJOA with considerable potential for clinical translation.

## Data Availability

The original contributions presented in the study are included in the article/**Supplementary Material**. Further inquiries can be directed to the corresponding author.
