# Supplementary material for: Intelligent GelMA microspheres co-loaded with BMSCs and Kartogenin for spatiotemporally controlled release and immunomodulatory regeneration of temporomandibular joint osteoarthritis
Source: Front Immunol. 2026 Sep 16;17:1921455. doi: 10.3389/fimmu.2026.1921455 (PMC13624099; doi:10.3389/fimmu.2026.1921455)
Supplement: Supplementary file 1 [file DataSheet1.docx]

Figure S1. Histological staining results of TMJ samples from rats subjected to MIA-induced modeling for four weeks in the preliminary study.
